# Mechanism of *Salvia miltiorrhiza Bge*. for the Treatment of Ischemic Stroke Based on Bioinformatics and Network Pharmacology

**DOI:** 10.1155/2022/1767421

**Published:** 2022-09-12

**Authors:** Jiaqi Wu, Ming Li, Ang Li, Xunming Ji

**Affiliations:** ^1^Beijing Advanced Innovation Center for Big Data-Based Precision Medicine, School of Biological Science and Medical Engineering, Beihang University, Beijing, China; ^2^China-America Institute of Neuroscience, Xuanwu Hospital, Capital Medical University, Beijing, China; ^3^Department of Biomedical Engineering, Columbia University, New York, NY, USA

## Abstract

**Methods:**

In this study, SymMap was used to screen the 50 bioactive scored components and 65 putative targets of *Salvia miltiorrhiza Bge.,* and their targets were standardized using the UniProt platform. The disease targets related to stroke were collected by comparative toxicogenomics database (CTD), GeneCards, and quantitative structure-activity relationships-TargetNet (QSAR-TargetNet). Thereafter, the protein-protein interaction (PPI) network was constructed using the STRING platform and visualized by Cytoscape (3.8.2) software. Then, the Metascape platform was used to analyze the Gene Ontology (GO) enrichment analysis and Kyoto Encyclopedia of Genes and Genomes (KEGG) pathway. Cytoscape (3.7.2) software was also used to construct the network of the “herb-component-target-pathway.” We found that Tanshinol B, Tanshinol A, Przewaquinone C, Tanshinone II, and other main components of *Salvia miltiorrhiza Bge.* may regulate neurotransmitters and neurological function. Therefore, we speculate *Salvia miltiorrhiza Bge.* has a neuroprotective effect. For further verification, potential core targets (STAT3, MMP2, ESR1, TERT, and MMP9 proteins) for ischemic stroke and core active ingredients (Tanshinol A, Tanshinol B, Tanshinone II A, and Przewaquinone C) for *Salvia miltiorrhiza Bge.* were further verified by molecular docking.

**Results:**

Our findings revealed that Tanshinol A, Tanshinol B, Tanshinone II A, and Przewaquinone C as the main component of *Salvia miltiorrhiza Bge.* may have a neuroprotective effect against ischemic stroke, which provides a new understanding for the development of therapies for the prevention and treatment of ischemic stroke.

## 1. Introduction

Stroke is one of the three leading causes of death in the world. Ischemic stroke is a common disease with a high incidence rate, mortality rate, and disability rate. It mainly occurs in the elderly, and it has a trend of becoming younger in recent years [1]. A large number of studies have shown that *Salvia miltiorrhiza Bge.* has unique advantages in the treatment of stroke, and can also improve symptoms of patients in the recovery period. In addition, it plays an irreplaceable role in improving patients' quality of life [2].

An earlier study showed that the experimental group was treated with *Salvia miltiorrhiza Bge.* compound injection and the control group treated with routine treatment, The total effective rates of the treatment group and the control group were 88.3% and 68.3% respectively. In the treatment group, the consciousness of comatose patients recovered, increased from 33.3% to 61.1%, and the incidence of side effects decreased [3].

The mechanism of Tanshinone I in treating ischemic stroke have been verified. Infarct volume and neurological deficit were assessed by 2, 3, 5-triphenyltetrazolium chloride (TTC) staining and Longa. Tanshinone I was found to reduce the volume of cerebral infarction induced by transient middle cerebral artery occlusion (tMCAO) in a dose-dependent manner. it was observed neuronal death in the hippocampus and cortex by detecting the changes of Neuronal nuclear antigen (NeuN) by Immunohistochemistry. It was found that pretreatment using Tanshinone I could improve the cell viability of HT-22 cells after oxygen-glucose deprivation (OGD) *in vitro* [4].

The percentage of CD4+CD25+FOXP3+ Treg cells in the peripheral blood of the MCAO group was increased with Cryptotanshinone treatment. The protein level of FOXP3 and the phosphorylation of STAT5 were recovered in the CD4+CD25+ Treg cells of the model group after being treated with Cryptotanshinone [5].

Although some experiments have proved that after ischemia-reperfusion, free radicals will be produced, leading to cell death. After middle cerebral artery occlusion, compared with the sham operation group, *Salvia miltiorrhiza Bge.* extract reduced the infarct area of the core, penumbra and subcutaneous, and the neurological deficit score was also decreased (*P* < 0.05) [6].

However, the above studies have laid a foundation for us to study the molecular mechanism of *Salvia miltiorrhiza Bge.* in alleviating ischemic penumbra. The pharmacological effect and molecular mechanism of *Salvia miltiorrhiza Bge.* on ischemic penumbra caused by ischemic stroke have not been fully elucidated [7]. We used a variety of public bioinformatics databases and pharmacological information resources to reveal the intrinsic pharmacological and potential beneficial effects of *Salvia miltiorrhiza Bge.* in the treatment of ischemic stroke through evaluation of pharmacological components, prediction of drug binding sensitive binding sites, molecular docking, network, and pathway analysis.

In this study, we followed the methods of Xu et al. [8] and explain the following problems:

1. According to the existing literature reports and network pharmacology knowledge, we constructed the pathway network formed by different pathological characteristics of ischemic stroke candidate biomarkers and selected components of *Salvia miltiorrhiza Bge.*
2. Based on their integration scores, 56 possible *Salvia miltiorrhiza Bge.* target proteins were selected and analyzed for GO and KEGG pathway enrichment, PPI interaction network of important proteins was also constructed. We constructed and analyzed the composite targeting pathway (CTP) network between the main components of *Salvia miltiorrhiza Bge.* and ischemic stroke.
3. The binding energies of four components of *Salvia miltiorrhiza Bge.* and seven ischemic stroke proteins were calculated by molecular docking method, especially potential active compounds (Tanshinol A, Tanshinol B, Tanshinone II A, and Przewaquinone C) and ischemic stroke related proteins (MMP2, STAT3, TERT, and ESR1), which has a good binding ability.

## 2. Results

### 2.1. Presumption of Candidate Chemical Compounds and Prognosis of Target Proteins

**Figure 1:**
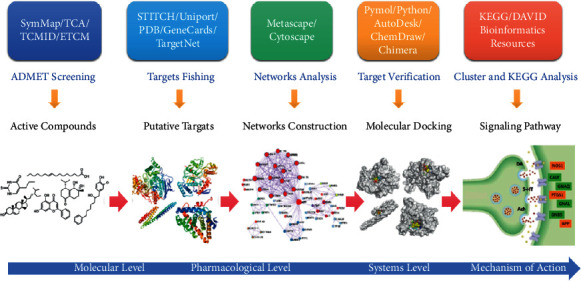
Network pharmacology for *Salvia miltiorrhiza Bge.* acting on stroke.

The potential pharmacological with biochemical mechanisms and the details behind each step of using systematic pharmacology to determine the role of *Salvia miltiorrhiza Bge.* in stroke are shown in Figure 1.

In order to further analyze the mechanism of *Salvia miltiorrhiza Bge.* in the treatment of stroke, the drug correlation and potential targets between stroke and *Salvia miltiorrhiza Bge.* were studied in more detail.

**Figure 2:**
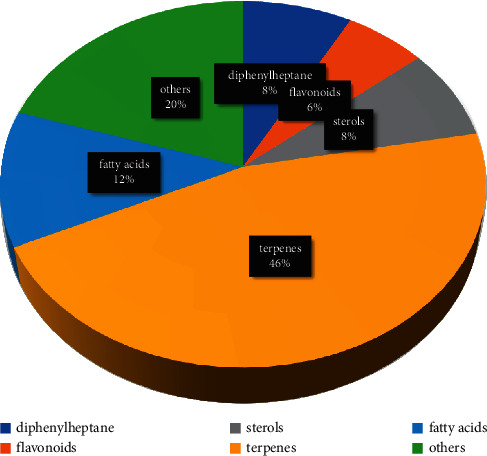
The classification candidate compounds of *Salvia miltiorrhiza Bge.*

Through the screening of the traditional Chinese medicine databases including the Traditional Chinese Medicine System Pharmacology database (TCMSP), the Traditional Chinese Medicine Integrated Database (TCMID), the Traditional Chinese Medicine Information Database (TCMID), and the Encyclopedia of Traditional Chinese Medicine database (ETCM). More than 50 active components in *Salvia miltiorrhiza Bge.* were found, which can be divided into six categories: terpenes, fatty acids, diphenyl heptane, sterols, flavonoids, and others, as shown in Figure 2.

Similarly, we take the union of the collected data sets and obeyed the rules described in Section 4.2 to obtain target proteins associated with ischemic stroke to build the datasets of ischemic stroke-related protein as a disease protein database. Among them, the established rules are that there is a high docking probability and the protein may have a good binding with the compound. A total of 7408 target proteins with high reliability were collected.

Furthermore, we selected 26 components with a value of oral bioavailability (OB) greater than 36% and a value of drug-likeness (DL) greater than 0.2 (Table S1).

Among these target proteins, 109 key target proteins were screened, and their docking chemical structure, target protein type, binding potential percentage, protein UniProt ID, and common name were summarized, as shown in Table S2.

**Figure 3:**
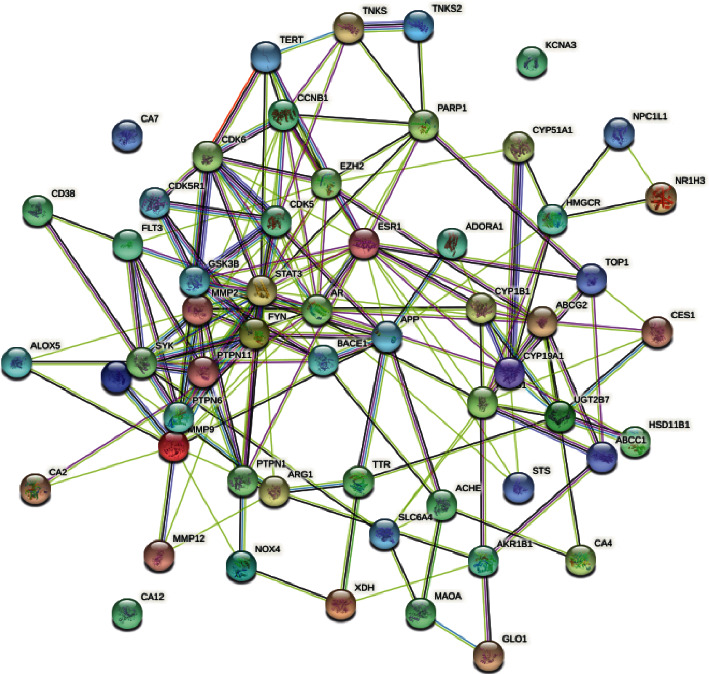
PPI network of common target between stroke and traditional Chinese medicine Danshen.

In addition, the construction and description of PPI networks are based on different classes of compounds and putative protein arrangements (Figure 3).

As shown in Figure 3, the PPI interaction has 56 nodes with 633 edges, of which the average node degree is 6.36, and after calculation, the average coefficient of its clustering is 0.438, *P* < 1.0*E* − 16, indicating that there is a significant difference between this group of interaction targets and random similar target combinations.

### 2.2. Exploration of *Salvia miltiorrhiza Bge*. Molecular Mechanism of Action

Based on their integration scores, 56 possible *Salvia miltiorrhiza Bge.* target proteins were selected and analyzed for GO and KEGG pathway enrichment. As shown in Tables S3 and S4, the filter rule is that the *P*-value cut-off value should be less than 0.01, 824 GO items of terpene and 48 items of KEGG pathway were identified. A total of 824 GO terms are included. The display of GO enrichment used bubble charts, as shown in Figures S2A–S2C, the *P*-values are arranged in descending order, and more important response to biological regulation (GO:0065007), cellular process (GO:0009987), metabolic process (GO:0008152). In addition, it also contains some genes (ACHE, PARP1, AKR1B1, APP, ARG1, GSK3B, MMP2, ABCC1, PTPN1, PTPN11, SLC6A4, STAT3, BACE1, NOX4, MMP9, and MMP12) in the cell's response to nitrogen compounds. The target pathways of *Salvia miltiorrhiza Bge.* and stroke are including axon guidance, steroid hormone biosynthesis, nitrogen metabolism, metabolic pathways, Serotonergic synapse, and Alzheimer`s disease (Figure S2D).

Therefore, we further confirmed that *Salvia miltiorrhiza Bge.* plays an important regulatory part in a series of nervous system diseases in especially ischemic stroke.

### 2.3. Molecular Mechanism of *Salvia miltiorrhiza Bge*

We collected the proteins related to stroke, mainly through Comparative toxicogenomics database (CTD) and Genecards database, and collected 6227 protein entries by combining the above data. There are 66 related proteins related to the main components of *Salvia miltiorrhiza Bge.*, among which 56 target proteins related to ischemic stroke were found, the overlap between the target proteins of *Salvia miltiorrhiza Bge.* major components and the target proteins related to ischemic stroke after clinical detection reflect the potential role of *Salvia miltiorrhiza Bge.* major components in the treatment of ischemic stroke.

### 2.4. Construction and Analysis of the Comprehensive Network Model of Main Components of *Salvia miltiorrhiza Bge*. and Ischemic Stroke

**Figure 4:**
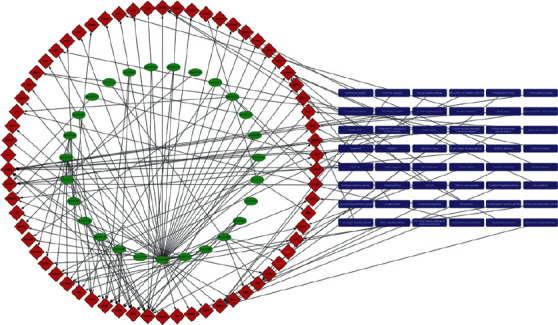
Construction and analysis of the composite targeting pathway (CTP) network between the main components of *Salvia miltiorrhiza Bge.* and ischemic stroke. (The details of Figure 4 can be downloaded from the supplementary file.)

*Salvia miltiorrhiza Bge.* complex targeted pathway disease (CTPD) network contains 87 nodes and 180 edges. To get a deeper understanding of the molecular mechanism of the neuroprotective mechanism of *Salvia miltiorrhiza Bge.*, we studied the complex targeting pathway of the main components of *Salvia miltiorrhiza Bge*. The network of “active ingredient target pathway” is shown in Figure 4. Fifteen signaling pathways related to the occurrence and development of ischemic stroke in KEGG enrichment are selected to show the pathway of *Salvia miltiorrhiza Bge.* on the target. The 56 major active components may act on 109 targets and play a role in 15 signaling pathways, which indicates that *Salvia miltiorrhiza Bge.* may inhibit the occurrence and development of ischemic stroke through multiple components, multiple targets and multiple pathways. The elliptical nodes with red border represent 13 ischemic stroke-related proteins and 4 key proteins. What's more, clustering and topology methods are used to identify individual differences and similarities between different protein targets (Figure S3).

Good blood-brain barrier permeability, pharmacokinetics, and water solubility play an important role in drug development. In order to better explore the mechanism of action and possible targets of the main components of *Salvia miltiorrhiza Bge.*, we selected the compounds that satisfied the mechanism of action and screening criteria and selected the common targets (CDK6, CCNB1, STAT3, MMP9, MMP2). Among these target proteins, Fyn and TTR are the overlapping nodes of ischemic stroke-related proteins, which have important value for future research.

### 2.5. Molecular Docking

**Figure 5:**
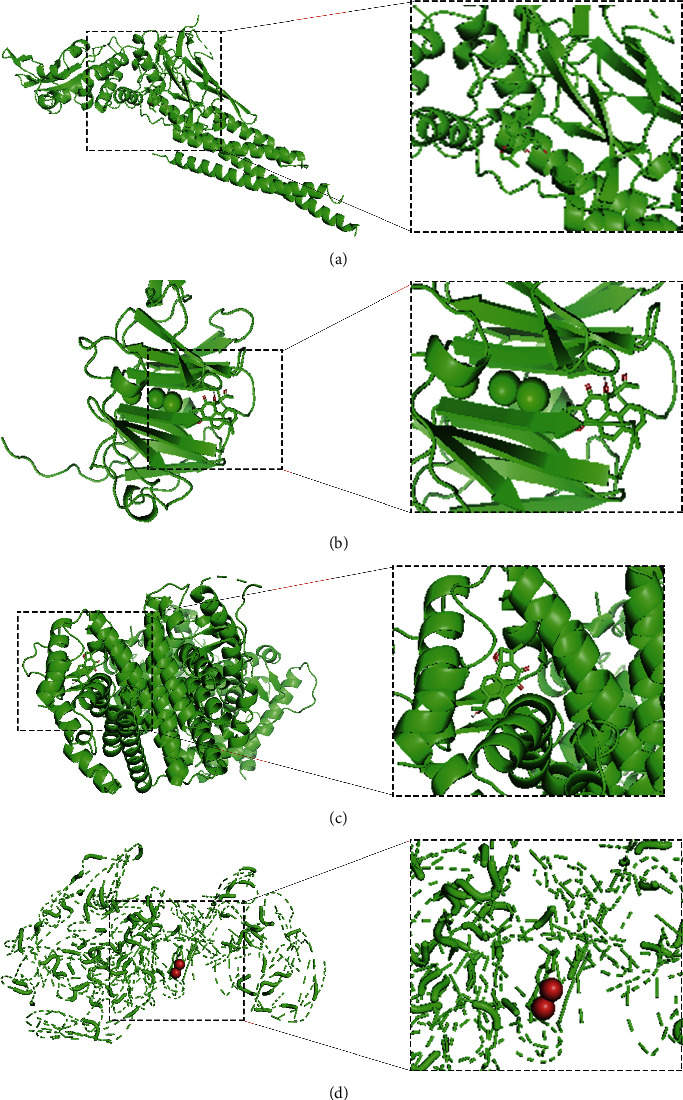
Three-dimensional schematic diagram of the molecular docking model and active site of the main components of *Salvia miltiorrhiza Bge*. with ischemic stroke. (a) Tanshinol B in the protein STAT3 (binding energy: −6.78 KJ/mol) (b) Tanshinol A in the protein MMP2 (binding energy: −9.14 KJ/mol). (c) Przewaquinone C in the protein ESR1 (binding energy: −8.52 KJ/mol). (d) Tanshinone II A in the protein TERT (binding energy: −8.33 KJ/mol).

According to the above analysis, it can be preliminarily concluded that *Salvia miltiorrhiza Bge.* has a certain effect on ischemic stroke. In order to further detect and verify the interaction of the selected active components of *Salvia miltiorrhiza Bge.* with key proteins, molecular docking of the above proteins and drug targets can be carried out. Using PyMOL software, Discovery studio 4.5 client, and Autodock tools 1.5.6 software, the interaction between potential active compounds (Tanshinol A, Tanshinol B, Tanshinone II A and Przewaquinone C) and ischemic stroke related proteins (MMP2, STAT3, TERT, and ESR1) was studied (Figure 5). The lower the binding energy, the stronger the binding ability. Van der Waals force, hydrogen bond, and aromatic accumulation were detected that plays an important role between the active center of targeted proteins and the target compounds (Figure 5).

## 3. Discussion

Traditional Chinese medicine has been proved by a large number of experiments to be a compound of multiple effective compounds. Through a variety of components, it can correspond to multiple proteins of the human body, so as to treat complex diseases. It is of great significance in the history of world pharmacology, especially in the history of herbal medicine.

Tanshinone is the main fat-soluble pharmacological component of *Salvia miltiorrhiza Bge. Salvia miltiorrhiza Bge.* is a famous traditional Chinese medicine for the treatment of cerebrovascular diseases, including ischemic stroke [9].

Amounts of experiments indicated that *Salvia miltiorrhiza Bge.* can reduce the possibility of ischemic stroke.

In transient middle cerebral artery occlusion (tMCAO) models, Tanshinone I (TSN I) was found to dose-dependently decrease mice's cerebral infarct volume induced in vivo. Compared with the cerebral ischemia-reperfusion group, the infarct volume significantly decreased in the 25 mg/kg TSN I intervention group, the number of NeuN-positive neurons was markedly increased in the 7 days of the TSN I intervention group, the protein levels of PI3K and *β*-catenin, as well as p-PDK1/PDK1, p-AKT/AKT, and p-GSK3*β*/GSK3*β* protein ratios was significantly increased in the TSN I pretreatment groups [10]. PI3K/AKT signaling pathway plays a pivotal role in the survival of neurons following cerebral ischemic injury [11]. TSN I can inhibit A beta aggregation, disaggregate A beta fibers, and reduce A beta-induced cell toxicity by the effects of antioxidation and anti-acetylcholinesterase in addition to promoting neurogenesis through increasing Wnt-3, p-GSK-3beta and beta-catenin immunoreactivities [12, 13].

*In vitro*, mainly using Neuro-2A cells cultured in Dulbecco's modified Eagle's medium and supplemented with 10% fetal bovine serum at 37°C and humidified in a 5% CO_2_ atmosphere and divided into different groups treated in different ways, pretreatment with Tanshinone I could increase cell viability of HT-22 cell following oxygen-glucose deprivation (OGD) [10]. The effect of Tanshinone IIA on cellular viability results showed that the viability was significantly increased in the Tanshinone IIA treatment group groups compared with the operation group (*P* < 0.05) [14]. the PI3K/mTOR signaling pathway was activated after treatment with Tanshinone IIA by detecting the expression of GLUT1 and HIF-1a and the ratio of p-AKT/AKT, p-mTOR/mTOR, and p-HER3/HER3 in cell model using western blotting analysis [14]. mTOR is a downstream molecule in the PI3K/AKT signaling pathway, and a previous study also found that activation of mTOR contributes to protection against cerebral ischemia-reperfusion injury [15].

This prompted us to study the possible active components, target proteins, and potential protein-drug mechanisms of *Salvia miltiorrhiza Bge.* and its main active ingredients in the treatment of ischemic stroke by integrating the methods of neurobiology, traditional Chinese medicine pharmacology and computational pharmacology, with the classical pharmacokinetic and models conducted by molecular docking.

The active components and targets of *Salvia miltiorrhiza Bge.* were collected through TCMSP and STITCH database, and the targets of ischemic stroke were downloaded from the website; Based on string platform data, the protein-protein interaction network (PPI) of *Salvia miltiorrhiza Bge*. on the common target of ischemic stroke was constructed by using Cystoscope software; The key genes of the signaling pathway (hub gene) were screened by using cytohubba in Cystoscope software, and the topological network of the target was constructed; The network diagram of “active ingredient target pathway” was drawn; Schrodinger software was used to docking the active components with the target.

The “active component target pathway” network showed that there were 56 main active components and 38 main targets in *Salvia miltiorrhiza Bge.*, which were related to 20 signaling pathways.

Furthermore, KEGG and GO annotation can also be used to infer the specific function and potential role of these proteins. Through the construction of the PPI network, the function of key proteins was predicted.

Cell components are mainly enriched in the cell, intracellular, cytoplasm, membrane-bounded organelle, intracellular organelle, intracellular membrane-bounded organelle, membrane, plasma membrane, nucleus, and so on, as shown in Figures S2A–S2C and Table S3.

**Figure 6:**
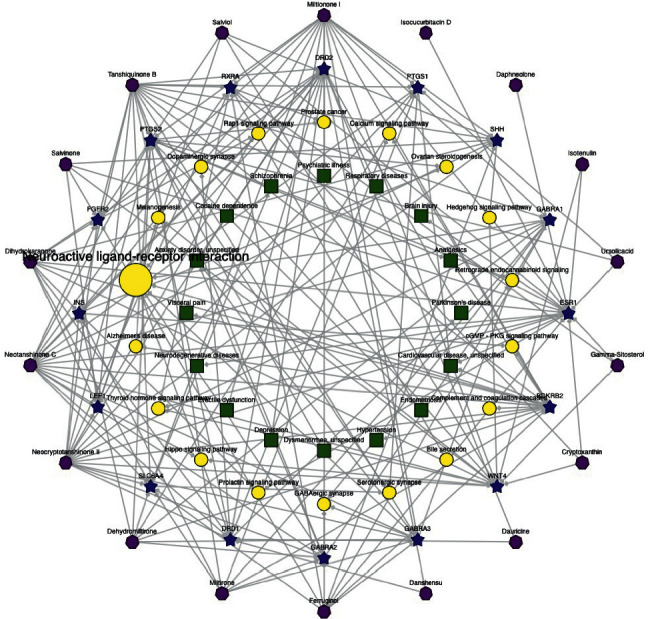
Ingredient-target-pathway/disease network of *Salvia miltiorrhiza Bge*. The purple hexagon represents constituent absorbed into blood, the blue five-pointed star represents drug target, the yellow circle represents the KEGG pathway, the red square represents online mendelian inheritance in man (OMIM) disease, and the green square represents comparative toxicogenomics database (CTD) disease.

According to the above interaction network and annotated map, the main components of *Salvia miltiorrhiza Bge.* play an important role in regulating the neural rehabilitation, the formation and repair of neurons, and signal transmission of ischemic stroke (Figure 6).

Figure 6 shows that the ingredient-target-pathway/disease network of *Salvia miltiorrhiza Bge.* in the network users can only exhibit those targets with no fewer than six linking compounds.

The network graph is drawn based on the known and predicted candidate target proteins with scores not smaller than 20 of each query ingredient of *Salvia miltiorrhiza Bge.* And in the “Simplified network view,” only significantly enriched KEGG pathways and Online Mendelian Inheritance in Man (OMIM) or Therapeutic Target Database (TTD) disease enrichment with adjusted *P*-value smaller than 0.05 are shown.

The purple hexagon represents the constituent absorbed into the blood, The blue five-pointed star represents drug target, The yellow circle represents the KEGG pathway, the red square represents OMIM disease, and the green square represents CTD disease.

We can find out *Salvia miltiorrhiza Bge.* related with brain injury, vascular disease, neurodegenerative diseases and also depression, heart failure, and visceral pain. Component targeting pathway/disease network reveals the possible association between ischemic stroke and other comorbidities and the theoretical basis of network pathway level.

KEGG enrichment analysis involved 48 signaling pathways, and 15 KEGG pathways were screened by the number of genes more than 4, as shown in Figure S2D and Table S4, Metabolic pathways, Pathways in cancer, MicroRNAs in cancer, Proteoglycans in cancer, Steroid hormone biosynthesis, Insulin resistance, Alzheimer's disease, Nitrogen metabolism, Bile secretion, Serotonergic synapse, Natural killer cell-mediated cytotoxicity, and so on.

As shown in Table S5 and Figure S4, the hub target of the interaction between the active components and ischemic stroke may be the signal transducer and activator of transcription 3 (STAT3), Estrogen Receptor 1 (ESR1), Androgen Receptor(AR), G2/Mitotic-Specific Cyclin-B1 (CCNB1), Enhancer Of Zeste 2 Polycomb Repressive Complex 2 (EZH2), Cyclin-dependent kinase 6 (CDK6), Matrix metalloproteinase-9 (MMP9), matrix metallopeptidase 2 (MMP2), poly(ADP-ribose) polymerase (PARP), Matrix Metallopeptidase 1 (MMP1), Telomerase reverse Transcriptase (TERT), Glycogen synthase kinase-3 beta (GSK3B), and Cyclin-dependent-like kinase 5 (CDK5).

There is still great research value for the four more meaningful stroke-related proteins (STAT3, MMP2, ESR1, TERT) in this study, and pointed out the direction for our further research.

On the molecular level, STAT3 found in this experiment might have a neuroprotective effect against cerebral ischemic injury through IL-6R-mediated by phosphorylation of signal transducer and activator of transcription 3 (STAT3), and finally induced neuronal cell death via a decrease in manganese-superoxide dismutase (Mn-SOD) [16].

Matrix metalloproteinases (MMPs) are zinc-containing endopeptidases that digest the components of the extracellular matrix that form the basal lamina surrounding the neurovascular unit, Several studies have suggested a strong relationship between the induction of MMP-2 and brain edema following cerebral ischemic injury, Matrix metalloproteinases (MMPs) and tissue inhibitors to metalloproteinases (TIMPs) modulate capillary permeability [17].

MMP-mediated proteolysis of neurovascular matrix may also interfere with homeostatic signals between different cell types in the neurovascular unit. Resting matrix signaling via integrins is vital for normal cell function. Disruption of extracellular matrix by MMPs can induce anoikic in neurons and cerebral endothelial cells [18, 19], indicating that MMP-2 may be an important target to prevent edema complications following cerebral ischemia.

Estrogen receptor alpha (ESR1), not beta, is a critical link in estradiol-mediated protection against brain injury, through knockout animal models experiments [20]. Telomerase reverse transcriptase (TERT) is one of the most important proteins related to leukocyte telomere length (LTL) [21], Which has been considered a crucial factor that associated with age-associated diseases [22], and the specific molecular biological mechanism is still worth studying.

Based on the network analysis of active components of *Salvia miltiorrhiza Bge.* and ischemic stroke, it is concluded that Tanshinol B, Tanshinol A, Przewaquinone C, Tanshinone II A of *Salvia miltiorrhiza Bge.* have good binding effect on the key proteins of ischemic stroke, such as STAT3, MMP2, ESR1, TERT. A large number of main components of *Salvia miltiorrhiza Bge.* have good molecular docking fraction, and they are highly matched with key proteins. It is of great significance to further test and discuss its specific functions and properties.

## 4. Materials and Methods

### 4.1. Compound Database of *Salvia miltiorrhiza Bge*. Building

In this study, we collected the chemical composition information of *Salvia miltiorrhiza Bge.* from SymMap (https://www.symmap.org/) [23], which is an comprehensive database covering the datasheet from the Traditional Chinese Medicine System Pharmacology (TCMSP) database (https://www.tcmsp-e.com/) [24], the Traditional Chinese Medicine Integrated Database (TCMID) [25] the Traditional Chinese Medicine Information Database (TCMID) [26], and the Encyclopedia of Traditional Chinese Medicine (ETCM; https://www.tcmip.cn/ETCM/index.php/Home/Index/) database [27]. Take the absorption, distribution, metabolism, excretion and toxicity of *Salvia miltiorrhiza Bge.* and Metabolism, excretion, and toxicity for ischemic stroke as the oral bioavailability (OB) standard to evaluate and screen the comprehensive drug similarity classification and pharmacokinetic characteristics of *Salvia miltiorrhiza Bge.* (OB ≥ 30%), drug-likeness (DL ≥ 0.18) assessment and blood-brain barrier (BBB) ≥ −0.3 respectively. In addition, the compounds of two-dimensional or three-dimensional (2D/3D) structure, also canonical smiles, and PubChem ID calibrated database with PubChem (https://pubchem.ncbi.nlm.nih.gov/).

### 4.2. Ischemic Stroke-Associated Target Proteins and Putative *Salvia miltiorrhiza Bge*. Target Protein Screening

Ischemic stroke-related protein targets were detected by the comparative toxicogenomics database (CTD; https://ctdbase.org/; inference score >1) [28] and the GeneCards database (https://www.genecards.org/) [29], we selected stroke related protein targets in CTD higher than 1 and targets in GeneCards higher than 0 in order to avoid the loss of putative targets for *Salvia miltiorrhiza Bge.*

Compounds were then scored by the Lipinski Rule of 5, and the putative ischemic stroke-related target protein screening, which was filtered conditionally from the quantitative structure-activity relationships-TargetNet (QSAR-TargetNet: https://targetnet.scbdd.com) [30]. The ischemic stroke-related proteins and putative targets of candidate *Salvia miltiorrhiza Bge.* compounds were verified by their unique UniProtKB ID and names in the UniProt database (https://www.uniprot.org/) [17].

### 4.3. Gene Ontology (GO) and Kyoto Encyclopedia of Genes and Genomes (KEGG) Pathway Enrichment and Network Constructions

Metascape (https://metascape.org/) [31] combines a GO [32] and KEGG [33] pathway enrichment analysis search to leverage over 40 independent knowledge bases. The GO/KEGG pathway enrichment terms of the proteins with a *P*-value less than 0.01 were regarded as significant interest targets.

To elucidate the pathogenesis of ischemic stroke and the mechanism of *Salvia miltiorrhiza Bge.*, the compound-target interaction (CT), compound-target-pathway (CTP), and compound-target-pathway-disease (CTPD) network were constructed.

The topology parameters are used to analyze, and the subnetworks of bottleneck nodes are filtered and visualized by Cytoscape 3.6.0 software (Institute for Systems Biology, Seattle, WA, USA; https://www.cytoscape.org/) [34]. Protein-protein interactions (PPI) were set up on Metascape and STRING (https://string-db.org/) [35] databases and also visualized using Cytoscape by cluster analysis.

### 4.4. Molecular Docking

The binding ability, site, and interaction between compounds and target proteins were analyzed by classical molecular dynamics using AutoDockTools-1.5.6, Pymol 2.3, and Discovery Studio 4.5 Client [36, 37]. The 3D chemical structure of the candidate Danshen compounds is derived from PubChem and energy minimization employed in ChemBioDraw 3D. However, the crystal structures of putative Danshen targets were got from the Protein Data Bank (https://www.pdb.org/) and decorated by removing the ligands and water motifs, revising it and optimizing the mutation sites, and adding hydrogen by the Pymol 2.3 and UCSF Chimera 1.14rc software.

## 5. Conclusions

In this study, we used network pharmacology and molecular docking methods to study the mechanism of ischemic stroke.

We found that as a traditional Chinese medicine, *Salvia miltiorrhiza Bge.* has multiple targets and multiple pathways, showing the overall performance.

For complex diseases with multiple causes and factors, such as stroke, compound drugs may be a better curative effect. Compound drugs have an effect of multiple targets and biological pathways.

However, our experiment found that a large number of genes have not been verified, which can also provide information for the application of *Salvia miltiorrhiza Bge.* in the treatment of hemorrhage or ischemic stroke.

*Salvia miltiorrhiza Bge.*, particularly the main chemical components, not only in the synthesis, release of neurotransmitters during transmission, or the formation and neuronal plasticity, there is a protective effect.

The main components of *Salvia miltiorrhiza Bge.* may be well combined with stroke-related proteins, which contributes to the new development in theory and the development of new drugs against ischemic stroke.

## Data Availability

The data used to support the findings of this study are included within the supplementary information file(s).

## Supplementary Materials

Supplementary Materials Figure S1: Venn diagram shows of target protein and major component binding protein of *Salvia miltiorrhiza Bge.* in ischemic stroke. Figure S2A: the cellular component in Gene Ontology (GO) of the top 20 putative target proteins of main component of *Salvia miltiorrhiza Bge.* (The horizontal axis represents the name of the cell component; the vertical axis represents the number of enrichments to this pathway.) Figure S2B: the molecular function in Gene Ontology (GO) of the top 20 putative target proteins of main component of *Salvia miltiorrhiza Bge.* (The horizontal axis represents the name of the molecular function; the vertical axis represents the number of enrichments to this pathway.) Figure S2C: the biological process in Gene Ontology (GO) of the top 20 putative target proteins of main component of *Salvia miltiorrhiza Bge.* (The horizontal axis represents the name of the biological process; the vertical axis represents the number of enrichments to this pathway.) Figure S2D: pathway enrichment analysis of KEGG pathway of the top 20 putative target proteins of main component of *Salvia miltiorrhiza Bge.* Figure S3: analysis of PPI protein interaction network between ischemic stroke and main components of *Salvia miltiorrhiza Bge.* Figure S4: hub target of the interaction between the active components and ischemic stroke. Table S1: results of 26 candidate high score compounds of *Salvia miltiorrhiza Bge.* Table S2: results of 103 putative high score target proteins of *Salvia miltiorrhiza Bge.* Table S3: results of 946 GO terms enrichment in *Salvia miltiorrhiza Bge.* Table S4: results of 48 KEGG terms enrichments in *Salvia miltiorrhiza Bge.* Table S5: characteristics of HUB gene in topological network.

## References

[1] Kalkonde Y. V., Alladi S., Kaul S., Hachinski V. (2018). Stroke prevention strategies in the developing world. *Stroke*.

[2] Fei Y.-X., Wang S.-Q., Yang L.-J. (2017). Salvia miltiorrhiza bunge (danshen) extract attenuates permanent cerebral ischemia through inhibiting platelet activation in rats. *Journal of Ethnopharmacology*.

[3] Hu Q. (1998). Observation on therapeutic efficacy of TCM in treating hemorrhagic stroke. *Chinese Journal of Integrated Traditional and Western Medicine in Intensive and Critical Care*.

[4] Jl A., Fw A., Peng S. A., Zx A., Yj C., Bing C. (2021). A network-based method for mechanistic investigation and neuroprotective effect on treatment of Tanshinone I against ischemic stroke in mouse. *Journal of Ethnopharmacology*.

[5] (2022). Cryptotanshinone possesses therapeutic effects on ischaemic stroke through regulating STAT5 in a rat model. https://pubmed.ncbi.nlm.nih.gov/33915069/.

[6] Ghasemloo E., Rahnema M., Bigdeli M. R. (2016). The neuroprotective effect of hydroalcoholic extract of salviaofficinalis on infract volume and neurologic deficitsin rat ischemicstroke model. https://jsmj.ajums.ac.ir/article_47405.html?lang=en.

[7] Mirzaei H., Momeni F., Saadatpour L. (2018). MicroRNA: relevance to stroke diagnosis, prognosis, and therapy. *Journal of Cellular Physiology*.

[8] Xu J., Wang F., Guo J. (2020). Pharmacological mechanisms underlying the neuroprotective effects of alpinia oxyphylla miq. On alzheimer’s disease. *International Journal of Molecular Sciences*.

[9] Lam B. Y. H., Lo A. C. Y., Sun X., Luo H. W., Chung S. K., Sucher N. J. (2003). Neuroprotective effects of tanshinones in transient focal cerebral ischemia in mice. *Phytomedicine*.

[10] Liu J., Wang F., Sheng P., Xia Z., Jiang Y., Yan B. C. (2021). A network-based method for mechanistic investigation and neuroprotective effect on treatment of Tanshinone I against ischemic stroke in mouse. *Journal of Ethnopharmacology*.

[11] Zhao E. Y., Cai L., Efendizade A., Ding Y. (2016). The role of akt (protein kinase B) and protein kinase C in ischemia-reperfusion injury. *Neurological Research: An Interdisciplinary Quarterly Journal*.

[12] Chen B. H., Park J. H., Cho J. H. (2016). Tanshinone I enhances neurogenesis in the mouse hippocampal dentate gyrus via increasing wnt-3, phosphorylated glycogen synthase kinase-3*β* and *β*-catenin immunoreactivities. *Neurochemical Research*.

[13] Zhou Y., Li W., Xu L., Chen L. (2011). In Salvia miltiorrhiza, phenolic acids possess protective properties against amyloid *β*-induced cytotoxicity, and tanshinones act as acetylcholinesterase inhibitors. *Environmental Toxicology and Pharmacology*.

[14] Wang J., Tong H., Wang X., Wang X., Wang Y. (2020). Tanshinone IIA alleviates the damage of neurocytes by targeting GLUT1 in ischaemia reperfusion model (*in vivo* and *in vitro* experiments). *Folia Neuropathologica*.

[15] Fuglesteg B. N., Tiron C., Jonassen A. K., Mjøs O. D., Ytrehus K. (2009). Pretreatment with Insulin before ischaemia reduces infarct size in langendorff-perfused rat hearts. *Acta Physiologica*.

[16] Jung J. E., Kim G. S., Chan P. H. (2011). Neuroprotection by interleukin-6 is mediated by signal transducer and activator of transcription 3 and antioxidative signaling in ischemic stroke. *Stroke*.

[17] Rosenberg G. A., Estrada E. Y., Dencoff J. E. (1998). Matrix metalloproteinases and TIMPs are associated with blood-brain barrier opening after reperfusion in rat brain. *Stroke*.

[18] Gu Z., Kaul M., Yan B. (2002). S-nitrosylation of matrix metalloproteinases: signaling pathway to neuronal cell death. *Science*.

[19] Lee S. R., Lo E. H. (2004). Induction of caspase-mediated cell death by matrix metalloproteinases in cerebral endothelial cells after hypoxia-reoxygenation. *Journal of Cerebral Blood Flow and Metabolism*.

[20] Dubal D. B., Zhu H., Yu J. (2001). Estrogen receptor alpha, not beta, is a critical link in estradiol-mediated protection against brain injury. *Proceedings of the National Academy of Sciences of the United States of America*.

[21] Scarabino D., Peconi M., Pelliccia F., Corbo R. M. (2019). Analysis of the association between TERC and TERT genetic variation and leukocyte telomere length and human lifespan-A follow-up study. *Genes*.

[22] (2022). Leukocyte telomere length: A focus on cerebrovascular events. https://pubmed.ncbi.nlm.nih.gov/22530730/.

[23] Wu Y., Zhang F., Yang K. (2019). An integrative database of traditional Chinese medicine enhanced by symptom mapping. *Nucleic Acids Research*.

[24] Ru J., Li P., Wang J. (2014). A database of systems pharmacology for drug discovery from herbal medicines. *Journal of Cheminformatics*.

[25] Huang L., Xie D., Yu Y. (2018). TCMID 2.0: a comprehensive resource for TCM. *Nucleic Acids Research*.

[26] Xue R., Fang Z., Zhang M., Yi Z., Wen C., Shi T. (2012). TCMID: traditional Chinese medicine integrative database for herb molecular mechanism analysis. *Nucleic Acids Research*.

[27] Hai-Yu X., Yan-Qiong Z., Zhen-Ming L. (2018). ETCM: an encyclopaedia of traditional Chinese medicine. *Nuclc Acids Research*.

[28] Davis A. P., Murphy C. G., Johnson R. (2012). The comparative toxicogenomics database: update 2013. *Nucleic Acids Research*.

[29] Stelzer G., Rosen N., Plaschkes I. (2016). The GeneCards suite: from gene data mining to disease genome sequence analyses. *Current Protocols in Bioinformatics*.

[30] Wang N.-N., Zhu M.-F., Shan A.-P., Yao Z.-J., Che Y.-J. (2016). TargetNet: a web service for predicting potential drug-target interaction profiling via multi-target SAR models. *Journal of Computer-Aided Molecular Design*.

[31] Zhou Y., Zhou B., Pache L. (2019). Metascape provides a biologist-oriented resource for the analysis of systems-level datasets. *Nature Communications*.

[32] TheGeneOntologyConsortium expansion of the gene Ontology knowledgebase and resources. *Nucleic Acids Research*.

[33] Ogata H., Goto S., Sato K., Fujibuchi W., Bono H., Kanehisa M. (1999). KEGG: Kyoto Encyclopedia of genes and Genomes. *Nucleic Acids Research*.

[34] Shannon P., Markiel A., Ozier O. (2003). Cytoscape: a software environment for integrated models of biomolecular interaction networks. *Genome Research*.

[35] Szklarczyk D., Gable A. L., Lyon D. (2018). String V11: protein-protein association networks with increased coverage, supporting functional Discovery in genome-wide experimental datasets. *Nucleic Acids Research*.

[36] Jiang L., Cao Q., Shin W. S., Eisenberg D. S. (2019). O4-01-01: inhibiting amyloid-beta cytotoxicity through its interaction with the cell SURFACE RECEPTOR lilrb2 BY structure-based design. *Alzheimer’s and Dementia*.

[37] Liu Y., Lai L., Ju Y., Liu C., Meng D. (2019). Chemical constituents and synergistic anti-gout studies on eurycoma longifolia and potential mechanisms evaluation based on systemic analysis approach. *Bioorganic Chemistry*.

