# Supplementary figures and images for: Mechanism of Salvia miltiorrhiza Bge. for the Treatment of Ischemic Stroke Based on Bioinformatics and Network Pharmacology
Source: Evid Based Complement Alternat Med. 2022 Sep 12;2022:1767421. doi: 10.1155/2022/1767421 (PMC9484879; doi:10.1155/2022/1767421)

**Stroke**

**Salvia**

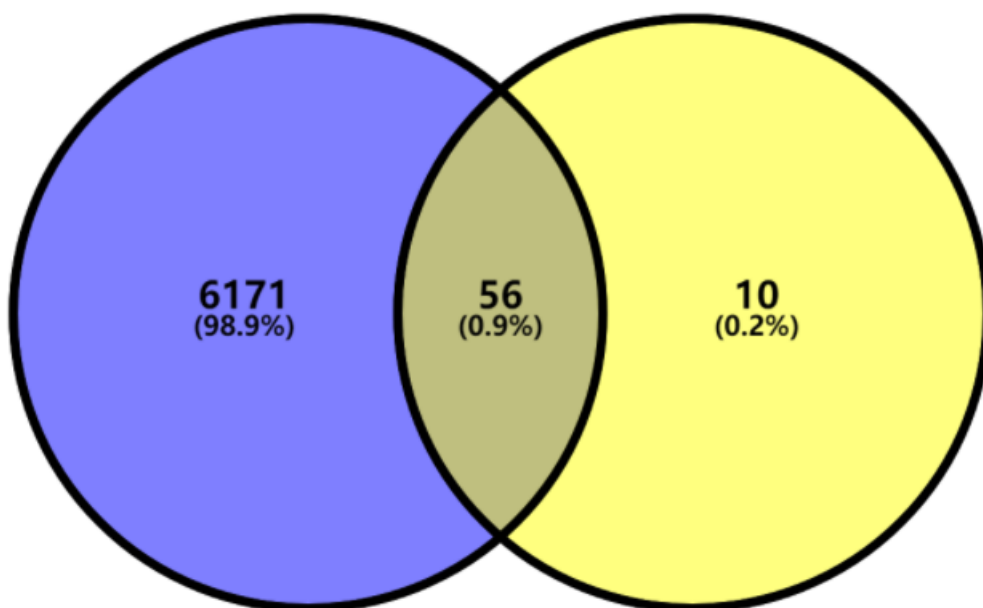

Supplement: Supplementary Materials — Figure S1: Venn diagram shows of target protein and major component binding protein of Salvia miltiorrhiza Bge. in ischemic stroke. Figure S2A: the cellular component in Gene Ontology (GO) of the top 20 putative target proteins of main component of Salvia miltiorrhiza Bge. (The horizontal axis represents the name of the cell component; the vertical axis represents the number of enrichments to this pathway.) Figure S2B: the molecular function in Gene Ontology (GO) of the top 20 putative target proteins of main component of Salvia miltiorrhiza Bge. (The horizontal axis represents the name of the molecular function; the vertical axis represents the number of enrichments to this pathway.) Figure S2C: the biological process in Gene Ontology (GO) of the top 20 putative target proteins of main component of Salvia miltiorrhiza Bge. (The horizontal axis represents the name of the biological process; the vertical axis represents the number of enrichments to this pathway.) Figure S2D: pathway enrichment analysis of KEGG pathway of the top 20 putative target proteins of main component of Salvia miltiorrhiza Bge. Figure S3: analysis of PPI protein interaction network between ischemic stroke and main components of Salvia miltiorrhiza Bge. Figure S4: hub target of the interaction between the active components and ischemic stroke. Table S1: results of 26 candidate high score compounds of Salvia miltiorrhiza Bge. Table S2: results of 103 putative high score target proteins of Salvia miltiorrhiza Bge. Table S3: results of 946 GO terms enrichment in Salvia miltiorrhiza Bge. Table S4: results of 48 KEGG terms enrichments in Salvia miltiorrhiza Bge. Table S5: characteristics of HUB gene in topological network. [file 1767421.f1.zip › 1767421.f1/FigureS1.pdf]

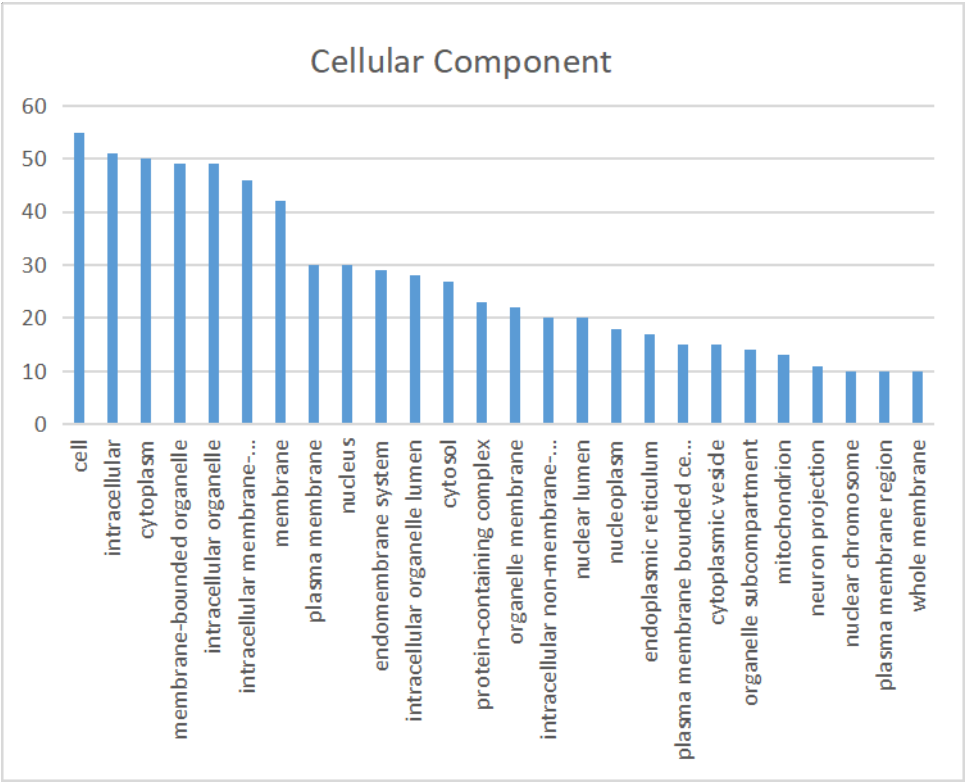

Supplement: Supplementary Materials — Figure S1: Venn diagram shows of target protein and major component binding protein of Salvia miltiorrhiza Bge. in ischemic stroke. Figure S2A: the cellular component in Gene Ontology (GO) of the top 20 putative target proteins of main component of Salvia miltiorrhiza Bge. (The horizontal axis represents the name of the cell component; the vertical axis represents the number of enrichments to this pathway.) Figure S2B: the molecular function in Gene Ontology (GO) of the top 20 putative target proteins of main component of Salvia miltiorrhiza Bge. (The horizontal axis represents the name of the molecular function; the vertical axis represents the number of enrichments to this pathway.) Figure S2C: the biological process in Gene Ontology (GO) of the top 20 putative target proteins of main component of Salvia miltiorrhiza Bge. (The horizontal axis represents the name of the biological process; the vertical axis represents the number of enrichments to this pathway.) Figure S2D: pathway enrichment analysis of KEGG pathway of the top 20 putative target proteins of main component of Salvia miltiorrhiza Bge. Figure S3: analysis of PPI protein interaction network between ischemic stroke and main components of Salvia miltiorrhiza Bge. Figure S4: hub target of the interaction between the active components and ischemic stroke. Table S1: results of 26 candidate high score compounds of Salvia miltiorrhiza Bge. Table S2: results of 103 putative high score target proteins of Salvia miltiorrhiza Bge. Table S3: results of 946 GO terms enrichment in Salvia miltiorrhiza Bge. Table S4: results of 48 KEGG terms enrichments in Salvia miltiorrhiza Bge. Table S5: characteristics of HUB gene in topological network. [file 1767421.f1.zip › 1767421.f1/FigureS2A.pdf]

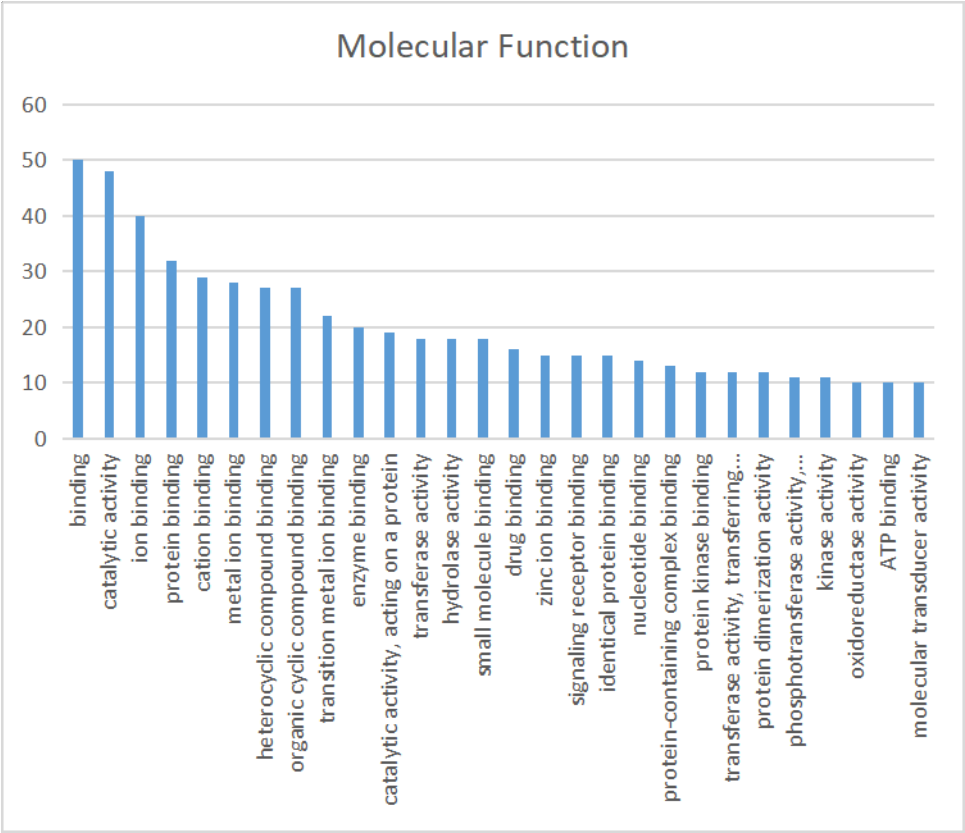

Supplement: Supplementary Materials — Figure S1: Venn diagram shows of target protein and major component binding protein of Salvia miltiorrhiza Bge. in ischemic stroke. Figure S2A: the cellular component in Gene Ontology (GO) of the top 20 putative target proteins of main component of Salvia miltiorrhiza Bge. (The horizontal axis represents the name of the cell component; the vertical axis represents the number of enrichments to this pathway.) Figure S2B: the molecular function in Gene Ontology (GO) of the top 20 putative target proteins of main component of Salvia miltiorrhiza Bge. (The horizontal axis represents the name of the molecular function; the vertical axis represents the number of enrichments to this pathway.) Figure S2C: the biological process in Gene Ontology (GO) of the top 20 putative target proteins of main component of Salvia miltiorrhiza Bge. (The horizontal axis represents the name of the biological process; the vertical axis represents the number of enrichments to this pathway.) Figure S2D: pathway enrichment analysis of KEGG pathway of the top 20 putative target proteins of main component of Salvia miltiorrhiza Bge. Figure S3: analysis of PPI protein interaction network between ischemic stroke and main components of Salvia miltiorrhiza Bge. Figure S4: hub target of the interaction between the active components and ischemic stroke. Table S1: results of 26 candidate high score compounds of Salvia miltiorrhiza Bge. Table S2: results of 103 putative high score target proteins of Salvia miltiorrhiza Bge. Table S3: results of 946 GO terms enrichment in Salvia miltiorrhiza Bge. Table S4: results of 48 KEGG terms enrichments in Salvia miltiorrhiza Bge. Table S5: characteristics of HUB gene in topological network. [file 1767421.f1.zip › 1767421.f1/FigureS2B.pdf]

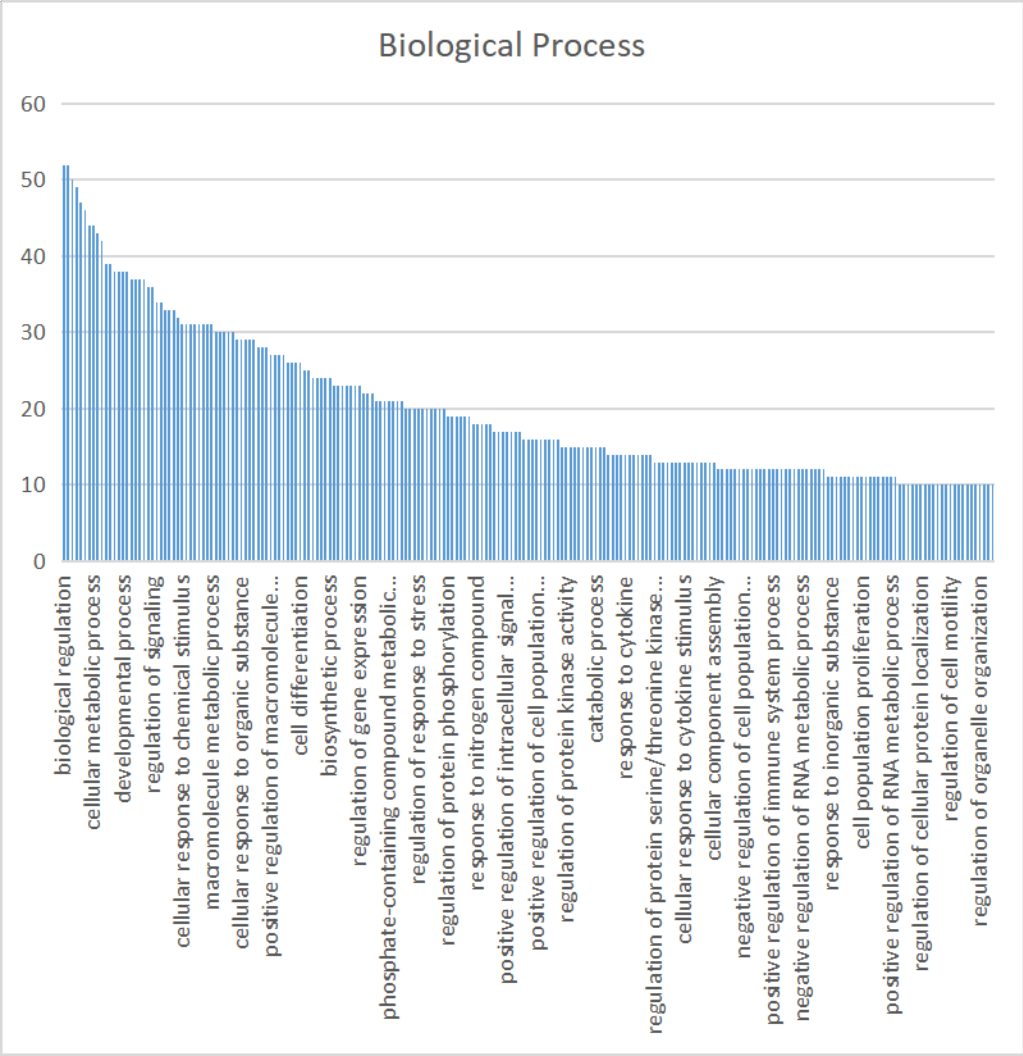

Supplement: Supplementary Materials — Figure S1: Venn diagram shows of target protein and major component binding protein of Salvia miltiorrhiza Bge. in ischemic stroke. Figure S2A: the cellular component in Gene Ontology (GO) of the top 20 putative target proteins of main component of Salvia miltiorrhiza Bge. (The horizontal axis represents the name of the cell component; the vertical axis represents the number of enrichments to this pathway.) Figure S2B: the molecular function in Gene Ontology (GO) of the top 20 putative target proteins of main component of Salvia miltiorrhiza Bge. (The horizontal axis represents the name of the molecular function; the vertical axis represents the number of enrichments to this pathway.) Figure S2C: the biological process in Gene Ontology (GO) of the top 20 putative target proteins of main component of Salvia miltiorrhiza Bge. (The horizontal axis represents the name of the biological process; the vertical axis represents the number of enrichments to this pathway.) Figure S2D: pathway enrichment analysis of KEGG pathway of the top 20 putative target proteins of main component of Salvia miltiorrhiza Bge. Figure S3: analysis of PPI protein interaction network between ischemic stroke and main components of Salvia miltiorrhiza Bge. Figure S4: hub target of the interaction between the active components and ischemic stroke. Table S1: results of 26 candidate high score compounds of Salvia miltiorrhiza Bge. Table S2: results of 103 putative high score target proteins of Salvia miltiorrhiza Bge. Table S3: results of 946 GO terms enrichment in Salvia miltiorrhiza Bge. Table S4: results of 48 KEGG terms enrichments in Salvia miltiorrhiza Bge. Table S5: characteristics of HUB gene in topological network. [file 1767421.f1.zip › 1767421.f1/FigureS2C.pdf]

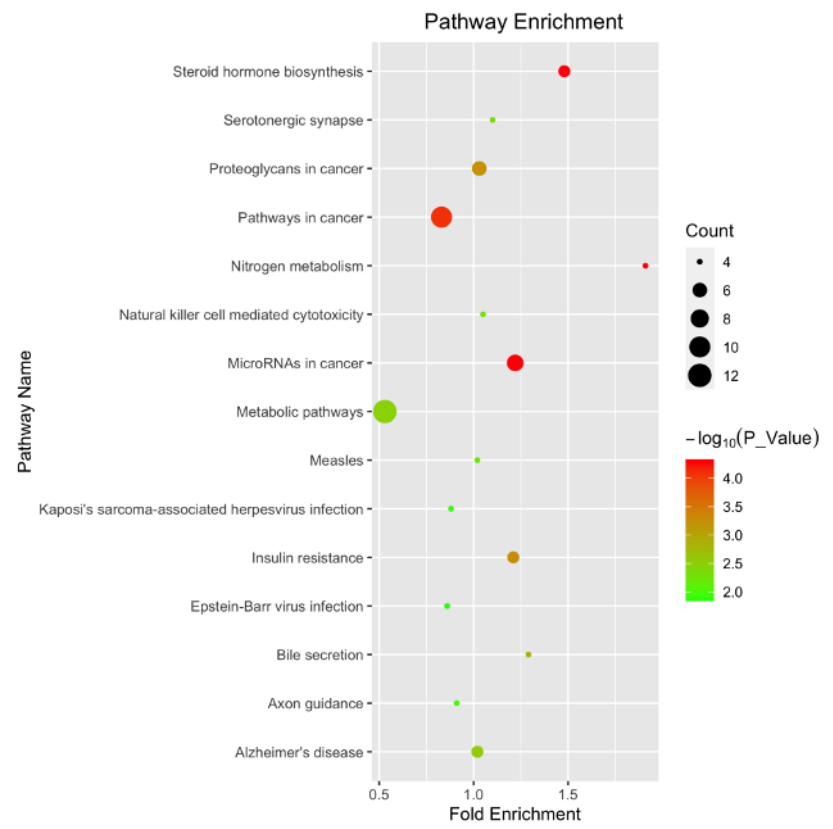

Supplement: Supplementary Materials — Figure S1: Venn diagram shows of target protein and major component binding protein of Salvia miltiorrhiza Bge. in ischemic stroke. Figure S2A: the cellular component in Gene Ontology (GO) of the top 20 putative target proteins of main component of Salvia miltiorrhiza Bge. (The horizontal axis represents the name of the cell component; the vertical axis represents the number of enrichments to this pathway.) Figure S2B: the molecular function in Gene Ontology (GO) of the top 20 putative target proteins of main component of Salvia miltiorrhiza Bge. (The horizontal axis represents the name of the molecular function; the vertical axis represents the number of enrichments to this pathway.) Figure S2C: the biological process in Gene Ontology (GO) of the top 20 putative target proteins of main component of Salvia miltiorrhiza Bge. (The horizontal axis represents the name of the biological process; the vertical axis represents the number of enrichments to this pathway.) Figure S2D: pathway enrichment analysis of KEGG pathway of the top 20 putative target proteins of main component of Salvia miltiorrhiza Bge. Figure S3: analysis of PPI protein interaction network between ischemic stroke and main components of Salvia miltiorrhiza Bge. Figure S4: hub target of the interaction between the active components and ischemic stroke. Table S1: results of 26 candidate high score compounds of Salvia miltiorrhiza Bge. Table S2: results of 103 putative high score target proteins of Salvia miltiorrhiza Bge. Table S3: results of 946 GO terms enrichment in Salvia miltiorrhiza Bge. Table S4: results of 48 KEGG terms enrichments in Salvia miltiorrhiza Bge. Table S5: characteristics of HUB gene in topological network. [file 1767421.f1.zip › 1767421.f1/FigureS2D.pdf]

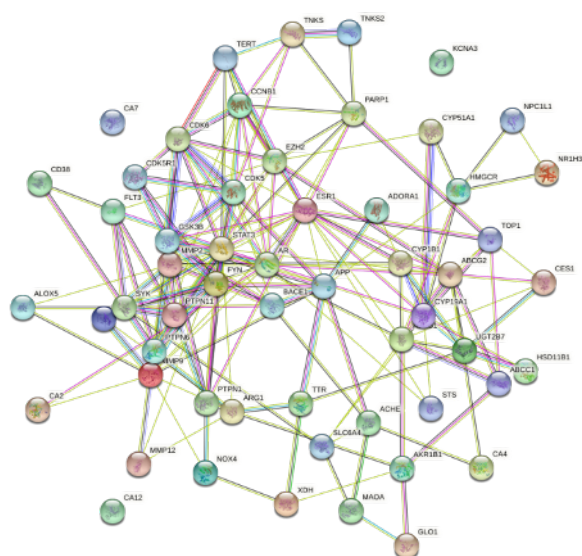

Supplement: Supplementary Materials — Figure S1: Venn diagram shows of target protein and major component binding protein of Salvia miltiorrhiza Bge. in ischemic stroke. Figure S2A: the cellular component in Gene Ontology (GO) of the top 20 putative target proteins of main component of Salvia miltiorrhiza Bge. (The horizontal axis represents the name of the cell component; the vertical axis represents the number of enrichments to this pathway.) Figure S2B: the molecular function in Gene Ontology (GO) of the top 20 putative target proteins of main component of Salvia miltiorrhiza Bge. (The horizontal axis represents the name of the molecular function; the vertical axis represents the number of enrichments to this pathway.) Figure S2C: the biological process in Gene Ontology (GO) of the top 20 putative target proteins of main component of Salvia miltiorrhiza Bge. (The horizontal axis represents the name of the biological process; the vertical axis represents the number of enrichments to this pathway.) Figure S2D: pathway enrichment analysis of KEGG pathway of the top 20 putative target proteins of main component of Salvia miltiorrhiza Bge. Figure S3: analysis of PPI protein interaction network between ischemic stroke and main components of Salvia miltiorrhiza Bge. Figure S4: hub target of the interaction between the active components and ischemic stroke. Table S1: results of 26 candidate high score compounds of Salvia miltiorrhiza Bge. Table S2: results of 103 putative high score target proteins of Salvia miltiorrhiza Bge. Table S3: results of 946 GO terms enrichment in Salvia miltiorrhiza Bge. Table S4: results of 48 KEGG terms enrichments in Salvia miltiorrhiza Bge. Table S5: characteristics of HUB gene in topological network. [file 1767421.f1.zip › 1767421.f1/FigureS3.pdf]

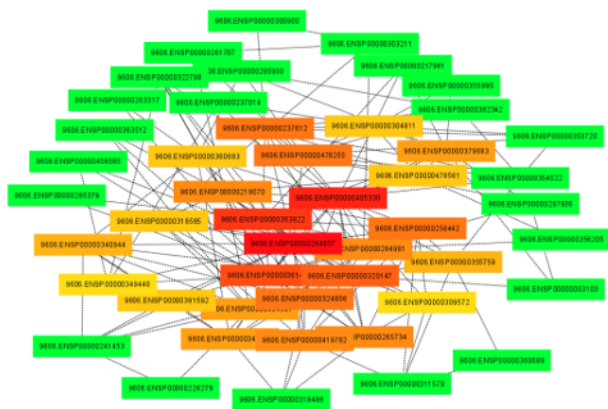

Supplement: Supplementary Materials — Figure S1: Venn diagram shows of target protein and major component binding protein of Salvia miltiorrhiza Bge. in ischemic stroke. Figure S2A: the cellular component in Gene Ontology (GO) of the top 20 putative target proteins of main component of Salvia miltiorrhiza Bge. (The horizontal axis represents the name of the cell component; the vertical axis represents the number of enrichments to this pathway.) Figure S2B: the molecular function in Gene Ontology (GO) of the top 20 putative target proteins of main component of Salvia miltiorrhiza Bge. (The horizontal axis represents the name of the molecular function; the vertical axis represents the number of enrichments to this pathway.) Figure S2C: the biological process in Gene Ontology (GO) of the top 20 putative target proteins of main component of Salvia miltiorrhiza Bge. (The horizontal axis represents the name of the biological process; the vertical axis represents the number of enrichments to this pathway.) Figure S2D: pathway enrichment analysis of KEGG pathway of the top 20 putative target proteins of main component of Salvia miltiorrhiza Bge. Figure S3: analysis of PPI protein interaction network between ischemic stroke and main components of Salvia miltiorrhiza Bge. Figure S4: hub target of the interaction between the active components and ischemic stroke. Table S1: results of 26 candidate high score compounds of Salvia miltiorrhiza Bge. Table S2: results of 103 putative high score target proteins of Salvia miltiorrhiza Bge. Table S3: results of 946 GO terms enrichment in Salvia miltiorrhiza Bge. Table S4: results of 48 KEGG terms enrichments in Salvia miltiorrhiza Bge. Table S5: characteristics of HUB gene in topological network. [file 1767421.f1.zip › 1767421.f1/FigureS4.pdf]
